# Gambling Harm-Minimisation Tools and Their Impact on Gambling Behaviour: A Review of the Empirical Evidence

**DOI:** 10.3390/ijerph21080998

**Published:** 2024-07-30

**Authors:** Ben J. Riley, Jane Oakes, Sharon Lawn

**Affiliations:** 1 College of Medicine and Public Health, Flinders University, Adelaide 5042, Australia; oakesejane@gmail.com (J.O.); sharon.lawn@flinders.edu.au (S.L.); 2 Southern Adelaide Local Health Network, Adelaide 5000, Australia

**Keywords:** gambling, problem gambling, harm reduction, prevention, effectiveness, consumer protection tools

## Abstract

The harms accompanying disordered gambling are well documented. Additionally, there is growing attention to the harms that arise from people who gamble heavily but do not meet the criteria for a gambling disorder. Accordingly, there has been an increasing interest in the effectiveness of consumer protection tools for consumers of gambling products. Subsequently, there is a need to properly evaluate the evidence for their effectiveness. This review aimed to conduct a narrative synthesis of empirical studies to identify gaps, weaknesses, and strengths in the existing evidence for the effectiveness of harm minimisation tools available to people who gamble. This review includes studies published between January 2015 to July 2022 and comprises 55 peer-reviewed studies for final synthesis. Findings reveal that while more research is needed to examine the effectiveness of active and passive consumer protection tools, uptake of tools is low in part because users view them as tools for individuals already experiencing gambling harm as opposed to protective tools for all users. Research is needed to determine effective ways of communicating the value of consumer protection tools for gambling.

## 1. Introduction

Gambling disorder is associated with harmful personal and social impacts such as job loss, family breakdown, incarceration, and suicide [1]. While there is extensive research examining treatments for individuals who develop gambling problems [2,3,4], a shift toward a more holistic multifaceted approach to gambling harm has widened the focus to consider opportunities for harm minimisation, early intervention, and prevention. The harms accompanying disordered gambling among people who gamble (hereafter referred to as “gamblers” [5]) and their significant others [6] are well documented. Additionally, there is growing attention paid to the harms that arise from people who gamble heavily and do not meet the criteria for a gambling disorder [7,8], generally referred to as “low-risk” and “moderate-risk” gamblers [9]. Interest in this group has arisen because, whilst the harms experienced by low- and moderate-risk gamblers may be less severe than those with gambling problems, there are a greater number of low- and moderate-risk gamblers; hence, the burden of harms is greater. Further, there is some evidence that subclinical gamblers experience greater psychopathology than healthy individuals. A study by Weinstock et al. [10] found that gamblers who did not meet the diagnostic threshold for gambling disorder but endorsed 2–3 gambling disorder criteria experienced greater psychological and psychosocial dysfunction than healthy individuals, and psychosocial impairment that was similar in intensity to individuals who met diagnostic criteria for mild-severity substance use disorder. Therefore, there is a considerable need and opportunity to recognise and reduce the harms experienced by gamblers across all risk groups. As such, there has been an increasing interest in the effectiveness of consumer protection tools for gamblers [11,12]. This is particularly important as few individuals experiencing gambling-related harm seek formal help. A study by Bijker et al. [13] highlighted that only 1 in 25 people with moderate-risk gambling and 1 in 5 people experiencing gambling problems had sought help for their gambling. Harm-minimisation tools may provide opportunities for harm reduction and the prevention of severe harm.

A range of harm-minimisation tools are available for gamblers. Voluntary self-exclusion programs allow gamblers to ban themselves from entering a specified gambling venue or venues, whether they be land-based [14] or online [15]. Provision of personalised feedback that informs gamblers of their expenditure or whether they have exceeded preset limits is provided by online [16,17] and land-based [18] operators. A recent study by Gainsbury et al. [19] examined the perceptions, motivators, and barriers to the use of consumer protector tools on internet gambling sites. Most gamblers surveyed were aware of the tools; however, usage was generally low, and tools were mostly used by individuals at risk of problem gambling. While harm-minimisation tools such as activity statements, deposit limits, and temporary self-exclusion are provided by most Internet gambling operators [12], a study examining 50 gambling websites found that there were inconsistencies in the tools offered [17]. In Australia, and many other jurisdictions, gambling operators’ duty of care to protect gamblers from gambling-related harm is stated in gambling legislation, although guidelines about how operators should achieve this are inconsistent. For instance, in Australia, some states are required to abide by mandated codes of practice enforceable by a regulatory body, while in others, the gambling industry develops its codes of practice and is self-regulatory [20]. In contrast, regulatory laws in Switzerland are stricter, with casinos required to make every effort to prevent gambling problems from arising before they become a serious issue: Swiss casinos have a mandate to impose involuntary exclusion orders on gamblers they believe are gambling beyond their means, and the exclusion orders are applied to all casinos across the country [20]. In France, the promotion of harm-minimisation tools for online gamblers is mandated, with online gambling operators required to verify players’ identity and display a warning banner on the risk of gambling along with information about available consumer protection tools [21].

Inconsistencies around the promotion and implementation of harm-minimisation tools by gambling operators may be due to the largely self-regulatory nature of the various codes of conduct. Alcohol experts have voiced similar concerns about self-regulatory measures and responsible drinking messages from the alcohol industry. As Fiedler et al. [22] note, some studies argue that due to the alcohol industry’s conflicting objectives, to encourage consumption in the service of generating profits whilst discouraging overconsumption, the alcohol industry purposefully produces responsible drinking campaigns for marketing purposes. The authors further argue that such campaigns are not only ineffective but may in themselves be harmful by reinforcing current drinking patterns [22]. Similarly, it has been argued that responsible gambling messages may act as “dark nudges” that both encourage gambling and increase gamblers’ perceived stigma [23].

Given the background presented, there is a need to evaluate the evidence for the effectiveness of available gambling harm-minimisation tools. To that end, the objectives of this review were (1) to evaluate the existing evidence on the effectiveness of harm-minimisation tools for gamblers with respect to their impact on gambling behaviour, and (2) to identify gaps, weaknesses, and strengths in the evidence to inform future research. A recent Australian study based on a national telephone survey of 15,000 respondents reported that 39.5% gambled only at land-based venues, 4.6% online only, and 12.8% gambled on both, and the risk of problematic gambling was greater among mixed-mode gamblers than the other two groups [24]. The present review therefore included harm-minimisation tools across land-based venues and online operators.

## 2. Materials and Methods

While it is important to identify and synthesise peer-reviewed research on a particular topic or phenomenon, identifying gaps in our understanding is necessary for developing a research agenda to advance knowledge [25]. This review, therefore, focuses on both summarising the evidence and synthesising gaps across the body of literature. We used a systematic approach modified from Otto et al. [26]. Specifically, this comprised a systemic literature search of peer-reviewed literature; a summary of existing areas of research focus identified in the literature; identification of gaps noted within existing research (as identified by the authors); identification of gaps across the body of research; consultation between subject matter experts in our research team to identify gaps not identified in the body of research.

### 2.1. Search Strategy

**Figure 1:**
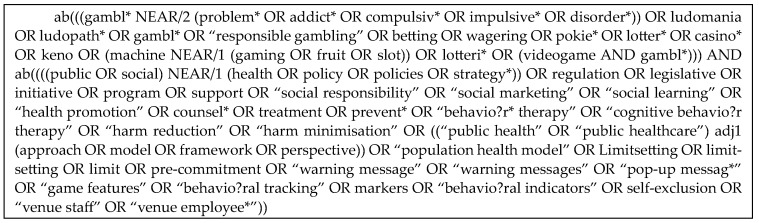
Search terms used for the ProQuest (Health & Medicine collection) database.

The search strategy was developed in consultation with a research librarian (see Figure 1). The peer-reviewed literature was sourced through searches of the electronic databases Medline, PsycINFO, Emcare and Proquest (Health & Medicine, Social Sciences Collection).

### 2.2. Inclusion Criteria

The emphasis for this review was on the available evidence relating to gambling harm-minimisation tools, rather than an extensive narrative summary of the available literature. For this reason, a replicable set of inclusion and exclusion criteria was used to determine if an article contained evidence of an empirical approach to evaluating gambling harm-minimisation tools. Table 1 lists the inclusion and exclusion criteria we adapted from Ladouceur et al. [27] in their synthesis of the empirical evidence on responsible gambling strategies. Ladouceur et al.’s [27] review targeted the period from 1962 to October 2015. Public health research on gambling harms expanded in the 2010s as researchers drew greater attention to the measurement and conceptualisation of gambling harms [28]. Given this, and the period covered by Ladouceur et al. [27], the search strategy for the current review targeted January 2015 to July 2022. The search was conducted on 4 July 2022 as part of a review and gap analysis of gambling harm reduction, funded by the Victorian Responsible Gambling Foundation [29]. While the original review addressed gambling harm reduction more broadly, the current view is focused on harm-minimisation tools available for gamblers.

**Figure 2:**
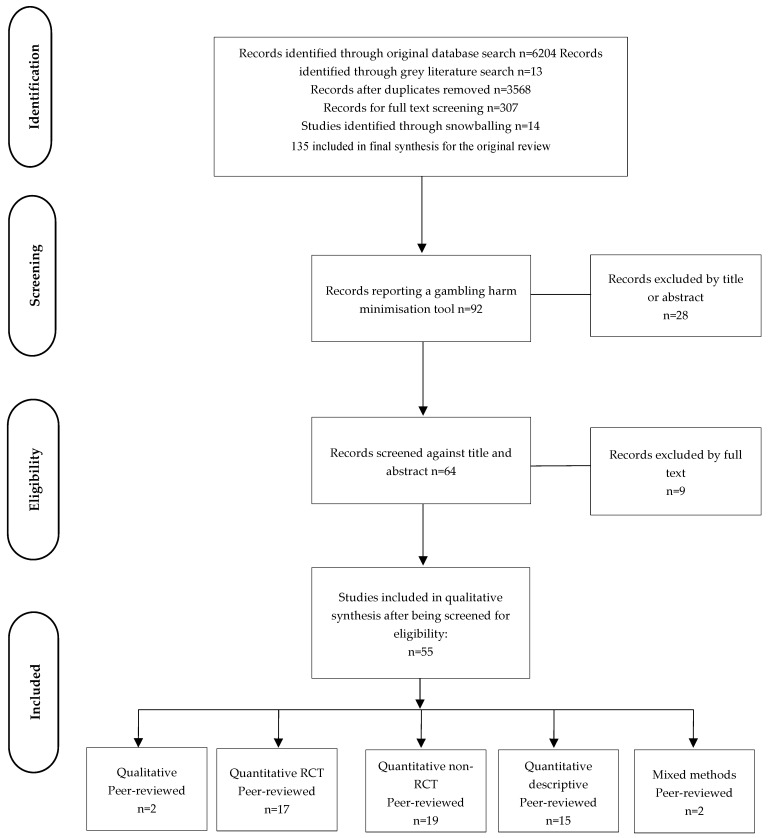
Flowchart of selection of articles according to PRISMA.

The search results were uploaded to Covidence [30] software for screening and data extraction. After the removal of duplicates, three reviewers (the authors) conducted the screening process. The inclusion and exclusion criteria for the current review were then applied to the 122 items acquired from the original search. This resulted in 57 peer-reviewed articles identified for the current review. The results of the search are presented in the PRISMA diagram presented in Figure 2.

The quality of the studies was assessed using the Mixed Methods Appraisal Tool (MMAT v. 18) for qualitative, quantitative randomised controlled trials, quantitative nonrandomised trials, quantitative descriptive, and mixed-method studies [31] (see Supplementary Material for the results of quality appraisals).

## 3. Results

We determined that all reviewed research was generally well conducted. The most frequently identified issue among randomised control trials was a failure to properly describe the randomisation process or whether assessors were blinded to the intervention. The most common issue among quantitative studies was that many did not account for confounders. The three qualitative studies were rated as high quality.

A range of harm-minimisation tools were reported across studies. Some tools require user buy-in (the user must actively sign up or register with the chosen harm-reduction tool to use it). Others are provided to gamblers without requiring them to opt-in. We made this distinction by categorising interventions as either active engagement (requiring user buy-in) or passive engagement. The 55 studies comprised eight categories of harm-minimisation tools: voluntary self-exclusion (n = 12), voluntary limit-setting (n = 11), pop-up messages (n = 9), passive personalised feedback (n = 8), active personalised behavioural feedback (n = 7), forced breaks (n = 4), third-party exclusion (n = 3), and speed of play (n = 1). The Hayer et al. [32] study included voluntary self-exclusion and third-party exclusion, so it was included in both categories. Table 2 presents an overview of the results.

### 3.1. Active Engagement Harm-Reduction Tools for Gamblers

#### 3.1.1. Voluntary Limit-Setting

Precommitment tools help gamblers stay in control of their gambling by allowing them to preset a limit for how much money they intend to gamble [33]. Several studies suggested that precommitment tools had some potential harm-reduction benefits. In a laboratory-based study, Brevers et al. [34] reported that financial risk-taking was reduced when participants had the opportunity to precommit to their forthcoming online gamble. Auer et al. [35] conducted an analysis of almost 50,000 gamblers and found that players classed as high-intensity gamblers decreased their gambling expenditure significantly more compared to gamblers who did not choose a deposit limit. However, only 1.31% of gamblers voluntarily used the precommitment tool. To increase the uptake of a precommitment tool, Heirene and Gainsbury [36] sent messages via email or account notifications to around 25,000 users of Australian online sports and racing gambling websites and compared message recipients against a control group who did not receive the messages. Whilst the impact was small, a greater number of gamblers who received the messages (0.71%) set a deposit limit compared to those who did not receive them (0.08%). Further, while only a small minority of gamblers used the precommitment tool, a reduction in gambling behaviour was observed among gamblers who set a deposit limit compared to non-limit-setters. In another study examining the use of prompting messages designed to increase the uptake of limit-setting tools, Ivanova et al. [33] sent messages to online gamblers either before registration, immediately after registration, or after their first deposit. They compared outcomes of message recipients with a control group. In contrast to Heirene and Gainsbury’s [36] findings, Ivanova et al. [33] found that prompts to set a deposit limit were not associated with a reduction in gambling behaviour, despite message recipients being more inclined to set limits.

Auer and Griffiths [37] demonstrated that it was possible to predict future limit-setting among online gamblers based on player behaviour. The most important variables predicting future limit-setting were players received feedback advising they had reached 80% of their global loss limit, personal monthly loss limit, the amount bet, theoretical loss (amount of bet multiplied by the house advantage across all games), and whether they had increased their limits in the past. These predictive analytics enable players with a high likelihood of changing their limits, to be proactively approached by gambling operators. Voluntary precommitment at the payment gateway level, effectively enabling gamblers to apply their budget setting on all gambling platforms they chose to play, was examined in a qualitative study by Lakew [38]. Respondents, most of whom did not see their gambling as harmful, viewed the tool as a first-line defence and reported that it helped them to plan their gambling activities.

In response to evidence indicating that some gamblers exceed their voluntarily set limits [39], particularly those with gambling problems [40], Rodda et al. [41] investigated the feasibility of a brief intervention delivered in gambling venues, to improve gamblers’ adherence to voluntary expenditure limits. Gamblers’ intentions to spend less in the 30 days after the intervention, compared to 30 days prior, were observed for the intervention group but not the control group. However, gamblers in both groups exceeded their set limits at the 30-day follow-up assessment. In another study, informative pop-up feedback messages aimed at assisting gamblers to adhere to their voluntary set limits were found to be associated with greater adherence to set limits [42], though this was not observed among gamblers who were assessed as highly financially focused (gambling for financial gain). Hollingshead et al. [43], however, found that personalised behavioural feedback did not improve limit adherence among gamblers playing a virtual electronic gaming machine (EGM) at a gambling venue.

#### 3.1.2. Exclusion

Exclusion can be voluntary, initiated by the gambler, which is typically referred to as voluntary self-exclusion (VSE). Additionally, it can be initiated without the agreement of the gambler, by a concerned third party, such as a family member or a gambling operator. This is typically referred to as a third-party exclusion (TPE).

Several studies have reported positive outcomes among gamblers who voluntarily self-exclude, though breaches of VSE are common and appear to be overlooked by gambling venue staff. McCormick et al. [44] reported reductions in problem gambling symptoms after 6 months of self-exclusion, though fewer reductions in symptoms were reported among the fifth of excluders who attempted to breach their VSE. Pickering et al. [45] analysed retrospective data concerning the experiences and outcomes of individuals enrolled in a centralised multivenue VSE program for up to 24 months. Approximately one-third breached the program by gambling in a nominated exclusion venue. The fear of embarrassment, if detected, was considered the main reason for contributing to compliance. The paperless system eased enrolment procedures, and the capacity to simultaneously exclude gamblers from multiple venues was considered the most helpful program feature.

The effectiveness of a 7-day temporary nonreducible VSE for at-risk online gamblers was investigated by Caillon et al. [46] using an experimental randomised controlled trial. A temporary self-exclusion on Internet gambling sites did not modify short-term gambling habits but did reduce gambling cravings and cognitive distortions. The findings suggest that a brief self-exclusion period may be effective for initiating change; however, the duration was too brief to modify longer-term gambling behaviour. In another study of the use of VSE among online gamblers, Catania and Griffiths [11] investigated whether the use of VSE is a reliable proxy measure for problem gambling, using data from an online gambling platform. One-fifth of the self-excluders reported having engaged with online gambling for less than 24 h, and half had less than 7 days of account registration before self-excluding. The results suggest that customers who self-exclude do so for a range of reasons, not necessarily due to experiencing a gambling problem. 

Hing, et al. [47] reported that self-exclusion had similar short-term outcomes to counselling alone with more rapid gambling abstinence; however, psychological treatment may be needed to sustain long-term change. Questions about the longer-term effectiveness of VSE as a harm-reduction strategy have similarly been raised by studies of online gamblers. Luquiens et al. [48] prospectively documented self-reported motives for self-exclusion among online poker players over 7 years. More than two-thirds of the gamblers returned to gambling after their first VSE. The authors suggested that self-exclusions should be accompanied by additional protective measures and treatment referral. In another study of online poker players, Luquiens, et al. [49] observed self-excluders 12 months following the expiry of their exclusion period and compared them to a matched control group. Overall, self-excluders spent less time and money gambling after the end of their VSE period than nonexcluders; however, there was no difference in money spent for heavier gamblers who applied their VSE period for a period of less than 3 months. Short-term exclusion, therefore, may not be an effective strategy for more involved gamblers.

Another study that examined gamblers’ return to gambling after either the expiration or withdrawal of their VSE evaluated the efficacy of an online tutorial created with the intent of reducing the risk of harm to those who return to gambling [50]. The benefits of self-exclusion were maintained at 6 and 12 months following the end of the exclusion period among gamblers who returned to gambling. There was no observed effect of the online tutorial. Yakovenko and Hodgins [51] compared an online and in-person self-management support intervention for gamblers who self-excluded. No differences were reported between the training conditions, with both groups experiencing a reduction in money and time spent gambling. However, none of the participants abstained from gambling completely for the duration of their voluntary exclusion period.

The importance of having VSE processes that are effective, easy for gamblers to use, and able to reach more people, was highlighted in studies by Yakovenko and Hodgins [50] and Pickering et al. [52]. Luquiens et al. [48] suggested that self-exclusion be accompanied by additional protective measures and treatment referral, and a VSE extension should be offered systematically outside the gambling environment. In general, improvements are needed at the entry and exit points of VSE registration. Findings from a study of casino excluders 8 years after exclusion found that VSE and TPE were associated with similarly reduced gambling behaviour [53].

Concerning TPE, a study by Lischer and Schwarz [54] explored the characteristics and experiences of gamblers excluded from casinos via VSE or TPE. Compared to VSE, gamblers who received TPE orders reported spending more time and money gambling prior to the TPE being imposed; however, TPE was not associated with a greater reduction in gambling when compared to cases of VSE. Further, during the exclusion period, many gamblers continued gambling by visiting casinos in neighbouring jurisdictions [53]. In a study of a TPE program based in Singapore, families reported financial and nonfinancial harms as motivation for applying for a TPE [55]. Participants who considered the TPE effective described a sense of relief and improved family finances and relationships.

Hayer et al. [56] analysed an administrative dataset of excluded gamblers from land-based gambling venues and reported that TPE accounted for only 1% of exclusions. Further, results revealed a lack of proper inspection, resulting in excluded players being able to gamble, and staff provided appropriate interventions to players with signs of problematic gambling in only 7% of cases. The authors suggested that the low level of compliance may be explained by an inherent conflict between economic interests and adequate protection of gamblers and concluded that operators of gaming venues rarely meet their statutory obligations regarding the exclusion of gamblers who are at risk of addiction.

#### 3.1.3. Active Personalised Behavioural Feedback

There is a growing interest in the use of personalised feedback tools for gamblers that provide them with information about their gambling activity. Seven studies investigated the use of providing voluntary personalised behavioural feedback to gamblers about their gambling activity. Four studies involved online gamblers only [16,56,57,58]; one involved a mix of online and land-based gamblers [59], and two involved land-based-only gamblers [18,60]. Three studies [16,57,58] used a comparison group (matched control). The method and content of feedback provided to gamblers differed among studies. Some provided push notifications when gamblers reached predetermined limits, while others provided a 3- or 6-month report of their expenditure.

Auer and Griffiths [16] provided some evidence that gamblers reduced their gambling when presented with a discrepancy between their perceived and actual expenditure. Online gamblers receiving personalised feedback spent significantly less time and money gambling compared to controls. A comparable study by Edson et al. [60] found that gamblers who screened positive on a brief problem gambling screen were more likely to unenroll from the tool, and more likely to respond negatively to notifications than users who screened negative. In a study of casino gamblers, gamblers who underreported their losses indicated that the feedback did not influence their subsequent gambling behaviour; however, player data for the following 3 months showed that their expenditure did, in fact, reduce [18].

Another feedback tool studied for online gamblers provided users with an assessment of their gambling based on their gambling data [58]. At-risk gamblers who opted to use the tool significantly reduced the amounts of money deposited and wagered compared to players who did not use the tool. Forsstrom et al. [59] examined a behavioural feedback tool among Norwegian online gamblers who had enrolled and then unenrolled; it was found that they had used the tool for approximately 7 days and there was no reduction in gambling.

### 3.2. Passive Engagement Harm-Reduction Tools for Gamblers

#### 3.2.1. Pop-Up Messages

We identified nine studies that examined the effectiveness of generic pop-up messages: four were conducted in laboratory settings and involved simulated EGMs [61,62,63,64], one involved a simulated roulette wheel [65], and four were conducted in real gambling venues with EGMs [66,67,68,69]. A range of messages were used and included warnings about harmful gambling [65] and messages that provided information and encouraged self-appraisal [61,66].

Overall, the evidence of the effectiveness of generic pop-up messages as an effective harm-reduction strategy is mixed. Several studies found them to be ineffective, with one study [61] showing that negative and self-evaluative messages were associated with an increase in gambling. Folkvord et al. [65] reported that pop-up pictorial and text warnings and helpline information failed to have a meaningful impact on EGM gambling. Ginley et al. [62] found that EGM gamblers who received warning messages while winning made the fewest number of spins and did not speed up their bet rate over the course of play as much as those not receiving pop-up messages. However, players who received warning messages while losing did not decrease their number of spins or rate of betting, suggesting that winning or losing while playing EGMs appears to impact the effectiveness of warning messages [62]. After demonstrating that both winning and losing streaks were associated with an increased speed of play, wagers, and gambling intensity while playing an EGM in a laboratory experiment, Harris and Parke [63] introduced self-appraisal pop-up messages to measure their impact during such streaks. The messages reduced the average speed of betting in the loss condition only, while both average stake size and betting intensity significantly increased following the pop-up message in both the win and loss conditions.

One study of the impact of EGM pop-up messages conducted in a real gambling venue found that warning messages had a small impact on reducing gambling consumption [66]. Another study conducted in situ involved researchers’ continuous observation of players’ gambling behaviour to examine the impact of EGM pop-up messages [68]. The results showed that pop-up messages were generally attended to but had little observable effect on gambler behaviour.

Despite the widespread promotion and availability of EGM jackpots, and evidence that EGM jackpots can intensify gambling behaviour [70], we identified just one study that examined the use of a harm-reduction pop-up message concerning EGM jackpots. Rockloff et al. [64] developed a novel jackpot “expiry” consumer protection approach that informs players after a set period that the jackpot had expired and could no longer be won by them. Gamblers presented with the relevant “expiry” message slowed their gambling significantly compared to gamblers who did not receive the message [64].

#### 3.2.2. Forced Breaks in Play

Another harm-minimisation strategy used by gambling operators is the use of mandatory breaks in play. Four studies were identified that examined the effectiveness of forced breaks in play in reducing gambling harm. Auer et al. [71] examined the effects of a forced 90 s break after 60 min of play among 7190 video lottery terminal gamblers in Norway. There was no observed reduction in gambling behaviour following the forced breaks. In 2015, a European online gambling operator introduced mandatory 60 min breaks in play that are automatically imposed after gamblers deposit at least 10 times over a 24 h period. Auer and Griffiths [72] examined the effects of this forced break intervention among 2021 British gamblers. The percentage of gamblers who stopped depositing money because of the forced break increased from 27% to 68% on the day of a play break, and the percentage of gamblers who stopped gambling because of the mandatory play break increased from 0.1% to 45% on the day of a play break.

In a laboratory-based study, Parke et al. [73] examined the effect of an imposed a 3 min break in play among 74 regular EGM gamblers while they played an online card gambling task. The intervention was effective at reducing the speed of wagering during periods of sustained losses. The effects of forced 3 and 8 min breaks in play on gambling cravings were examined by Blaszczynski et al. [74] in another laboratory-based study involving 141 university students playing an online simulated card gambling game. The results revealed that participants’ gambling cravings increased following the forced breaks, and the cravings were higher following the 8 min compared to the 3 min break.

#### 3.2.3. Speed of Play

There was just one study that investigated manipulation of the speed of play in terms of its effect on gambling behaviour. In an experiment using an online roulette wheel, Newall et al. [75] allocated 1002 experienced online gamblers to either a normal or slowed-down condition group. Limiting the speed of play led to a reduction in the amount of money gambled, despite participants spending more time gambling.

**Table 2:** Overview of results.

Category of Harm-Minimisation Tool (n = 55)	Number of Studies [References]	Summary of Results
Active engagement (n = 30)		
Voluntary self-exclusion	12 [11,32,44,45,46,47,48,49,50,51,52,53]	Positive outcomes for some gamblers, though breaches are common and are often overlooked by operators. The registration process should be easy to use and accompanied by additional protective measures.
Voluntary limit-setting	11 [33,34,35,36,37,38,39,40,41,42,43]	Potential harm-minimisation benefits for some gamblers; however, low uptake. Providing personalised behavioural feedback may increase the uptake of limit-setting tools.
Active personalised behavioural feedback	7 [16,18,56,57,58,59,60]	Some evidence that receiving feedback was associated with a reduction in gambling. There may be differences in how feedback is received by at-risk and nonrisky gamblers.
Passive engagement (n = 25)		
Pop-up messages	9 [61,62,63,64,65,66,67,68,69]	The evidence is mixed and inconclusive. Messages may be perceived differently depending on whether gamblers are winning or losing.
Passive personalised behavioural feedback	8 [76,77,78,79,80,81,82,83]	Some evidence that providing information about how gamblers’ behaviour compared to most other players led to a reduction in gambling. How feedback is received may differ between at-risk vs. nonrisky gamblers in terms of how it is provided, e.g., by letter or telephone.
Forced breaks	4 [70,71,72,73]	The evidence is mixed and inconclusive. Longer breaks (e.g., 60 min) vs. shorter breaks (e.g., 60 s) may be more effective.
Third-party exclusion	3 [32,54,55]	Not enough research to draw conclusions. However, like voluntary exclusion, studies reported that violations of exclusion orders were not uncommon.
Speed of play	1 [75]	One laboratory-based study reducing the speed of play was associated with a reduction in the amount of money gambled despite greater time spent gambling.

#### 3.2.4. Passive Personalised Behavioural Feedback

Studies reviewed in this section involve those that examined a behavioural feedback tool where the user was not required to opt in. This could be through experimental design where participants were randomised to each condition, for example, receiving feedback group or a nonfeedback control group, or the feedback tool was installed in the gambling device for all users, not requiring them to opt in. We identified eight such studies.

Two studies involved college students and used personalised normative feedback, provided as a once-off assessment of their gambling behaviour and information about how it compared to other students at the same university. Martens et al. [76] found that the personalised feedback condition reported fewer dollars gambled and fewer gambling-related problems than those in the assessment-only condition. Similarly, Neighbors et al. [77] found that students who received normative feedback about their gambling reported reduced gambling behaviour compared to their nonfeedback counterparts. Auer and Griffiths [78] also examined the effect of normative feedback among online gamblers. A pop-up message shown to gamblers who played 1000 consecutive games in an hour of play, informing them that very few other players exceed this number in a single gambling session, was associated with shorter gambling sessions when compared to non-normative generic RG messages.

McGivern et al. [79] examined the use of pop-up messages that provided casual gamblers with personalised feedback about their expenditure while gambling on an online roulette wheel. Expenditure messages were associated with a greater reduction in gambling when compared to other generic and control messages. Another study conducted in a laboratory involved gamblers playing a simulated EGM [80]. Personalised feedback comprised an onscreen quiz asking players if they knew how much they had spent, followed by a display of their actual spend, and a message congratulating them if they guessed correctly. The feedback was associated with improved accuracy of play estimates but did not reduce gamblers’ persistence or expenditure.

Another approach to the use of personalised feedback is to target high-consumption gamblers. Jonson et al. [81] randomly selected a sample of the top 5% of customers based on expenditure from a Norwegian gambling operator database. Gamblers were randomised to receive either personalised feedback about their gambling by telephone, letter, or no contact (control condition). Gamblers who received feedback reported greater reductions in their gambling compared to the control condition over a 3-month [81] and 1-year [82] follow-up period. A more recent analysis of the same data revealed that subtypes of gamblers may respond differently to the mode of contact. For example, high-consumption lottery gamblers responded better to feedback provided in the form of a letter, whereas high-consumption casino and sports gamblers responded better to feedback provided by telephone contact [83].

## 4. Discussion

Exclusion programs were the most studied harm-minimisation tool. The vast majority of these involved voluntary exclusion, with just three that involved nonvoluntary exclusion (TPE). There is a gap in the research on nonvoluntary exclusion programs. The literature indicates that breaches of exclusion orders are common and often go undetected by gambling venue staff, despite regulatory codes of conduct requiring staff, within reason, to enforce exclusion orders. This finding is consistent with the conclusions presented by Ladouceur et al. [27], who reported that while self-exclusion programs demonstrate some effectiveness, their limitations include low utilisation rates, breaching the agreement, and minimal evidence about long-term outcomes. There is a gap in the research into understanding the reasons why gamblers who have self-imposed exclusion orders return to gambling illegitimately. While there is some research that suggests that challenges concerning venue staff and gambler interactions stem from perceived conflicting roles [84,85], overall, there is a gap in research into how gambling venue staff respond to exclusion order breaches and why they rarely refer gamblers experiencing harm for professional help. Further, research is needed to better understand the most effective way for staff to respond to exclusion breaches, for example, the significance of timing when engaging with gamblers who have returned to gambling during an exclusion period has been raised previously [86].

Regarding online gambling, there are a variety of short-term VSE tools available and promoted by online gambling operators, such as exclusion periods of 1 to 7 days, several weeks, or months. There is a gap in evidence concerning the use and effectiveness of these tools and the harm-reduction outcomes in relation to the duration of the exclusion period. Understanding these evidence gaps is important given that the limited evidence available indicates that shorter-duration VSE programs are less effective, particularly among high-consumption gamblers.

Following exclusion programs, precommitment and voluntary personalised feedback were the next most studied tool. Overall, while precommitment tools are promoted by online betting companies as consumer protection measures in the same way as self-exclusion options, there is a gap in the evidence concerning the long-term effectiveness of these tools. In their review of evidence prior to 2015, Ladouceur et al. [27] reported inconsistencies in the effectiveness of limit-setting tools. Further research is therefore needed. Concerning opt-in behavioural feedback tools, the evidence suggests that those users who do choose to use consumer protection tools, such as receiving personalised feedback about their gambling behaviour, are gamblers who are at low risk and already gambling in a safe manner [60]. Further research is needed to examine why low-risk gamblers choose to use harm-reduction tools and why heavier gamblers choose not to. Further, it is important to understand the impact of providing gamblers feedback about their gambling behaviours. Intuitively, it appears helpful that gamblers be made aware of the consequences of their gambling. However, one study reported that when individuals with gambling problems are made aware of their financial losses, such insight can create significant distress as they realise the extent of what they have done [87]. There is a gap in the evidence concerning the attitudes of individuals experiencing gambling problems toward receiving behavioural feedback. Further, there is a gap in knowledge regarding the most effective ways to provide feedback to gamblers in a way that does not further augment the distress experienced by those with existing problems. In addition, future research should also consider which tools may in practice cause more harm.

Safe gambling messaging is another area that has received empirical attention, though, again, the evidence is mixed and there are gaps in knowledge. Most studies that examined the effectiveness of pop-up messages as a harm-reduction strategy involved EGMs or simulated online slots. There is a gap in the research on the effectiveness of safe gambling messages for other modes of gambling such as sports betting. There is a gap in the evidence of interventions designed to increase health literacy about gambling (e.g., statistics, probability, house-edge advantage) and gambling harms, particularly among target populations (e.g., young males, racial and ethnic minority groups). This is important, given emerging evidence that suggests that increasing gamblers’ understanding of complex concepts of gambling, such as return to player percentage by presenting the information in different ways, may influence their gambling behaviour [88,89]. Providing this information in contexts outside of gambling, that is, when people are not engaged in gambling, is an important consideration, as for some gamblers, critical thinking is suspended during play or battles with indecisiveness [87].

There has been growing criticism in recent years highlighting a conflict of interest between the economic objectives of the alcohol industry, which promotes encouraging alcohol consumption, and harm-reduction initiatives aimed at reducing alcohol-related harm, which typically means reducing consumption [90]. A recent review of the literature highlighted a distinct lack of evidence that industry-led harm-reduction initiatives within the alcohol industry do anything to reduce harmful drinking. The review concluded that the evidence strongly suggests that corporate social responsibility (CSR) initiatives are used by the industry to influence the framing of the nature of alcohol-related issues in line with industry interests [90], with some critics going so far as naming this conflict an “illusion of righteousness” [91] (p. 1). In a similar vein, researchers and policymakers need to question the fundamental notion that gambling operators (which are driven by profits) are suitably able to implement and regulate harm-reduction policies, including the promotion of consumer protection tools. A recent study examining the conflict between responsible gambling programs and the gambling industry’s financial interests [22] concluded that such CSR measures were ineffective due to conflicting interests. Specifically, these were the opposing interests between player protection and the profits driven by individuals with gambling problems that account for a large share of the total revenue that venues receive from gambling. Further, gambling profits are a significant source of government revenue. The authors suggest that CSR programs are effective only when business interests are not at stake, while otherwise mandatory rules with enforcement are required [22].

### 4.1. Future Research Directions and Gaps in the Evidence

Further research is needed to understand why gamblers, both at low risk and high risk, exceed their voluntary set limits. Research into strategies that may assist gamblers who set limits and adhere to them is in its infancy, and this line of enquiry should be expanded. For instance, the promotion of the recently developed low-risk gambling limits [92] could be examined with respect to gamblers sticking to set limits. Using player-tracking data to predict limit-setting behaviour of gamblers [37], along with other predictors of risk, is an area for further research. Additionally, the effectiveness of mandatory and opt-out limit-setting tools requires further examination. Overall, while precommitment tools, in the same way as the range of self-exclusion options offered, are widely promoted by online betting companies, there is a gap in the evidence concerning the long-term effectiveness of these tools.

Concerning gambling exclusion orders, there is very little research concerning TPE and, therefore, a gap in our understanding of their effectiveness and the attitudes of gamblers and their families towards them. There is a gap in the research concerning how gambling operators respond to being informed of exclusion violations, and operators’ perceptions of responsibility. Longitudinal research is needed to understand longer-term outcomes among gamblers post-exclusion. There is a gap in the evidence concerning the use and effectiveness of short-term VSE tools promoted by online gambling operators. This is important given that the limited evidence available indicates that shorter-duration VSE is less effective, particularly among heavier gamblers.

The provision of personalised feedback to gamblers about their gambling behaviour is a promising line of enquiry. However, given that most studies involve gamblers who have opted in to receive it, further research is needed to examine which gamblers opt in and their reasons for doing so, along with an exploration of why most gamblers do not, despite being aware of the tools. In addition, more research is needed on the effect of personalised feedback for at-risk gamblers and individuals with existing gambling problems. As has been suggested, the use of consumer protection tools appears to be voluntary by gamblers who might already be gambling in a safe manner [61]. Concerning the delivery of personalised feedback, more research is needed on the number and timing of messages, and types of feedback mechanisms, and longitudinal research is needed to determine whether any benefits are sustained over a longer time. The provision of personalised feedback on gambling activity is a promising line of enquiry and, overall, the evidence suggests that it could be a useful player protection tool, though few gamblers choose to use it. Research is needed to examine how to increase uptake, including opportunities for an opt-out, rather than opt-in, option. While the limited research on gamblers receiving unrequested personalised feedback suggests that it may be a helpful harm-reduction strategy, there is a gap in knowledge about how the information is best received (email, text message, letter) and the most effective types of information (normative comparison, gambling consumption, self-appraisal). Additionally, there is a gap in knowledge about the impact of unrequested feedback across levels of gambling consumption. Finally, more research is needed to examine the longer-term impacts of unrequested feedback, for example, the gambler’s level of distress, guilt, and shame.

More research is needed to better understand the impact of different types of pop-up messages (e.g., fear-based, self-appraisal, informative) among gamblers of varying characteristics and levels of problem gambling risk. There is a gap in the research on the effectiveness of safe gambling messages in the context of sports betting. Overall, more research is needed to refine the content for pop-up messaging, both in the context of online gambling and land-based gambling devices, to ensure it plays an effective role in consumer protection and harm minimisation.

Forced play breaks are offered by some gambling operators as a safer gambling tool. To date, the evidence is mixed. While gambling cravings were higher following an 8 min as opposed to a 3 min break, it appears that longer breaks, for example, 60 min as compared to 90 s, may be effective. More research is needed, particularly in the real-world context to understand the use of forced breaks as a safer gambling intervention. Future studies should aim to determine the optimal gambling session length before a forced break is introduced, and the most effective length of a forced break, and should involve different modes of gambling.

### 4.2. Limitations

This study provided the results of a literature review of gambling harm-minimisation tools and their effect on gambling behaviour. Despite the rigour applied throughout our screening process, there is the potential that some studies may have been inadvertently missed. Only peer-reviewed articles written in English were included. As such, there may have been relevant non-English peer-reviewed articles and studies in the grey literature that were not included. A more general limitation likely arises from considering gaps from the existing perspective. This perspective may have introduced an inherent bias by relying on the gaps and recommendations for further research that particular experts and research teams promoted as important. For example, this may account for the lack of research on specific subpopulations at greater risk of gambling harm, such as Indigenous, refugee, immigrant, and young adult male populations.

## 5. Conclusions

This literature review presents the results of a systematic approach to reviewing the current evidence on gambling harm-minimisation tools for gamblers. Notwithstanding the limitations acknowledged, this review provides a succinct summary of the relevant literature, evidence, and gaps in research concerning harm-minimisation tools for gamblers. More research is needed to examine the effectiveness of the range of available active and passive consumer protection tools offered by gambling operators. Research is also needed to determine more effective ways of communicating the value of consumer protection tools to users and their loved ones/families. The evidence indicates that the uptake of available tools is exceptionally low and suggests that one reason for this is that gamblers view them largely as tools developed for individuals with gambling problems, rather than tools to assist recreational gamblers to remain safe. Most studies included in this review concerned harm-minimisation tools that require active engagement. There is a need for a greater focus on how opt-out or mandatory tools can be better integrated to prevent harm without negatively affecting user experience. This would also serve to minimise harm for individuals who have developed a gambling disorder, in which impaired control is a central psychological construct of the condition [93]. The use of gambling consumer protection tools should be promoted for use by all gamblers. An analogy for this is seatbelts in cars. Seatbelts are recommended, in fact mandatory, in most jurisdictions for all drivers, because driving a car is associated with risk of harm. In other words, seatbelts are not perceived as protective tools for at-risk or dangerous drivers; rather, they are mandated for all drivers regardless of their risk status. Research is needed to examine how the use of gambling consumer protection tools can be communicated in a similar manner to gamblers, such as promoting their use by all gamblers once the tools are demonstrated to be effective.

## Figures and Tables

**Table 1:** Inclusion criteria.

Inclusion	Exclusion
Describes and evaluates a gambling harm-minimisation tool. Uses an empirical approach to evaluate the impact of the gambling harm-minimisation tool on gamblers’ behaviour. Conducted within a real or simulated/laboratory-based gambling environment with “real” gamblers or individuals affected by gambling-related harm. Published in peer-reviewed journal. Written in English.	Does not describe and evaluate a gambling harm-minimisation tool. Does not use an empirical approach to examine the impact of the gambling harm-minimisation tool on gamblers’ behaviour. Does not involve actual gamblers or individuals affected by gambling-related harm. Grey literature (technical reports, conference presentations, dissertations, books). Not written in English.

## Data Availability

Not applicable.

## Supplementary Materials

The following supporting information can be downloaded at: https://www.mdpi.com/article/10.3390/ijerph21080998/s1, Table S1: MMAT quality rating for qualitative studies; Table S2: MMAT quality rating for quantitative randomized controlled trials; Table S3: MMAT quality rating for quantitative nonrandomized trials; Table S4: MMAT quality rating for quantitative descriptive studies; Table S5: MMAT quality rating for mixed-methods studies.
