# Supplementary material for: Gambling Harm-Minimisation Tools and Their Impact on Gambling Behaviour: A Review of the Empirical Evidence
Source: Int J Environ Res Public Health. 2024 Jul 30;21(8):998. doi: 10.3390/ijerph21080998 (PMC11353816; doi:10.3390/ijerph21080998)
Supplement: Supplementary file 1 [file ijerph-21-00998-s001.zip › ijerph-3069220-supplementary.pdf]

Supplementary material. Quality ratings for included peer-reviewed studies

*MMAT v.18 quality rating: low = 1 to 2 stars; moderate = 3 stars; moderately high = 4 stars; high = 5 stars (Hong et al. 2018). *

Hong QN, Pluye P, Fabregues S, Bartlett G, Boardman F, Cargo M, et al. Mixed Methods Appraisal Tool Version 2018 (MMAT v.18) 2018. Available from: http://mixedmethodsappraisaltoolpublic.pbworks.com/w/file/fetch/127916259/MMAT_2018_criteria-manual_2018-08-01_ENG.pdf.

Table S1: MMAT Quality Rating for Qualitative Studies

**Author/Date**	**1.1. Is the qualitative approach appropriate to answer the research question?**	**1.2. Are the qualitative data collection methods adequate to address the research question?**	**1.3. Are the findings adequately derived from the data?**	**1.4. Is the interpretation of results sufficiently substantiated by data?**	**1.5. Is there coherence between qualitative data sources, collection, analysis and interpretation?**	**Overall Quality***
Goh et al. (2016)	Y	Y	Y	Y	Y	*****
Lakew (2022)	Y	Y	Y	Y	Y	*****

Y = Yes, N = No, C = Can’t Tell

*MMAT v.18 quality rating: low = 1 to 2 stars; moderate = 3 stars; moderately high = 4 stars; high = 5 stars (Hong et al. 2018).

Table S2: MMAT Quality Rating for Quantitative Randomized Controlled Trials

**Author/Date**	**2.1. Is randomization appropriately performed?**	**2.2. Are the groups comparable at baseline?**	**2.3. Are there complete outcome data?**	**2.4. Are outcome assessors blinded to the intervention provided?**	**2.5 Did the participants adhere to the assigned intervention?**	**Overall Quality** *
Blaszczynski et al. (2015)	C	C	Y	C	Y	**
Byrne et al. (2019)	C	C	Y	N	Y	**
Caillon et al. (2019)	C	Y	Y	C	Y	***
Folkvord et al. (2019)	C	C	Y	C	Y	**
Ginley et al. (2016)	C	C	Y	Y	C	**
Ivanova et al. (2019)	C	C	C	N	Y	*
Jonsson et al. (2019)	C	C	C	N	Y	*
Jonsson et al. (2020)	Y	Y	Y	Y	C	****
Jonsson et al. (2021)	Y	Y	Y	Y	C	****

Martens et al. (2015)	C	Y	C	C	Y	**
Neighbors et al. (2015)	Y	Y	N	Y	Y	****
Newall et al. (2022) (Impact of the…)	Y	Y	Y	C	C	***
Rodda et al. (2020)	Y	N	C	C	Y	**
Parke et al. (2019)	C	C	Y	C	C	*
Rockloff et a., (2015)	C	Y	Y	N	Y	***
Tabri et al. (2019)	C	Y	Y	N	Y	***
Yakovenko & Hodgins (2021)	Y	Y	Y	C	Y	****

Y = Yes, N = No, C = Can't Tell

*MMAT v.18 quality rating: low = 1 to 2 stars; moderate = 3 stars; moderately high = 4 stars; high = 5 stars (Hong et al. 2018). *

Table S3: MMAT Quality Rating for Quantitative Non-randomized Trials

**Author/Date**	**3.1. Are the participants representative of the target population?**	**3.2. Are measurements appropriate regarding both the outcome and intervention (or exposure)?**	**3.3. Are there complete outcome data?**	**3.4. Are the confounders accounted for in the design and analysis?**	**3.5. During the study period, is the intervention administered (or exposure occurred) as intended?**	**Overall Quality ***
Auer & Griffith (2015) (Testing normative…)	Y	Y	Y	Y	Y	*****
Auer & Griffin (2020) (The use of ….)	Y	N	Y	Y	Y	*****
Auer et al. (2018)	Y	Y	Y	N	Y	****
Auer et al. (2019) (The effects of a voluntary….)	Y	Y	Y	C	Y	****
Auer et al. (2019) (The effects of a mandatory…)	Y	Y	Y	Y	Y	*****

Brevers et al. (2016)	C	C	Y	C	Y	**
Gainsbury et al. (2015) (Determinl...)	Y	Y	Y	C	Y	****
Gainsbury et al. (2015) (Optimal...)	Y	Y	Y	C	Y	****
Harris & Parke (2015)	C	Y	Y	N	C	***
Hing et al. (2015)	Y	Y	Y	N	Y	****
Hollingshead et al. (2019)	Y	C	Y	N	Y	***
Luquiens et al. (2018)	Y	Y	C	N	Y	****
Luquiens et al. (2019)	Y	Y	Y	Y	Y	*****
McGivern et al. (2019)	Y	Y	Y	N	Y	****
Pickering et al. (2018)	Y	Y	C	C	N	**
Rockloff et al. (2013)	C	Y	Y	N	Y	***
Turner et al. (2021)	Y	Y	Y	C	Y	****
Wood et al. (2015)	Y	Y	Y	C	Y	****

Y = Yes, N = No, C = Can't Tell

*MMAT v.18 quality rating: low = 1 to 2 stars; moderate = 3 stars; moderately high = 4 stars; high = 5 stars (Hong et al. 2018). *

Table S4: MMAT Quality Rating for Quantitative Descriptive Studies

Author/Date	4.1. Is the sampling strategy relevant to address the research question?	4.2. Is the sample representative of the target population?	4.3. Are the measurements appropriate?	4.4. Is the risk of nonresponse bias low?	4.5. Is the statistical analysis appropriate to answer the research question?	Overall Quality *
Armstrong et al. (2018)	Y	C	Y	Y	Y	****
Auer et al. (2019)	Y	Y	Y	Y	Y	*****
Auer et al. (2020)	Y	Y	Y	Y	Y	*****
Auer & Griffiths (2022)	Y	Y	Y	Y	Y	*****
Broda et al. (2008)	Y	Y	Y	Y	Y	*****
Catania & Griffiths (2021)	Y	Y	C	Y	Y	****
Edson et al. (2021)	Y	C	Y	C	Y	***
Forsstrom et al. (2020) (Dropouts'...)	Y	Y	C	C	Y	***
Hayer et al. (2020)	Y	Y	Y	Y	Y	*****
Heirene et al. (2021)	Y	Y	Y	Y	Y	*****

Kotter et al. (2018)	Y	Y	Y	N	Y	****
Luquiens et al. (2019)	Y	Y	Y	Y	Y	*****
McCormick et al. (2018)	Y	Y	Y	Y	Y	*****
Palmer du Preez et al. (2016)	Y	Y	Y	Y	Y	*****
Pickering & Blaszczynski (2022)	Y	Y	Y	N	Y	****
Wohl et al. (2017)	Y	Y	Y	N	Y	****
Wood & Griffiths (2014)	Y	Y	Y	Y	Y	*****

Y = Yes, N = No, C = Can't Tell

*MMAT v.18 quality rating: low = 1 to 2 stars; moderate = 3 stars; moderately high = 4 stars; high = 5 stars (Hong et al. 2018). *

Table S5: MMAT Quality Rating for Mixed Methods Studies

Author/Date	5.1. Is there an adequate rationale for using a mixed methods design to address the research question?	5.2. Are the different components of the study effectively integrated to answer the research question?	5.3. Are the outputs of the integration of qualitative and quantitative components adequately interpreted?	5.4. Are divergences and inconsistencies between quantitative and qualitative results adequately addressed?	5.5. Do the different components of the study adhere to the quality criteria of each tradition of the methods involved?	Overall Quality *
Landon et al, (2917)	Y	Y	Y	Y	Y	*****
Lischer & Schwartz (2018)	Y	Y	C	C	N	**

Y = Yes, N = No, C = Can't Tell

*MMAT v.18 quality rating: low = 1 to 2 stars; moderate = 3 stars; moderately high = 4 stars; high = 5 stars (Hong et al. 2018). *
